# Survey of False-positive Reactivity of Latex Agglutination Test for Kala-azar (Katex) without Urine Sample Boiling Process in Autoimmune Patients

**Published:** 2017-06

**Authors:** Mohammad Amin GHATEE, Zahra KANANNEJAD, Iraj SHARIFI, Asma ASKARI, Mehdi BAMOROVAT

**Affiliations:** 1. Cellular and Molecular Research Center, Yasuj University of Medical Sciences, Yasuj, Iran; 2. Dept. of Immunology, School of Medicine, Shiraz University of Medical Sciences, Shiraz, Iran; 3. Leishmaniasis Research Center, Kerman University of Medical Sciences, Kerman, Iran; 4. Dept. of Pathobiology, Faculty of Veterinary Medicine, Shahid Bahonar University of Kerman, Kerman, Iran

**Keywords:** False positivity, KAtex, Rheumatoid factor, Autoimmune patients

## Abstract

**Background:**

Latex agglutination test for Kala-azar (KAtex) is an easy, inexpensive, and field-applicable antigen detection test. However, the main drawback of this method is the boiling step applied to remove false positivity of the test. This study was conducted to survey false positivity results of latex agglutination test for KAtex without boiling process in urine of some autoimmune patients.

**Methods:**

Ninety-two urine samples from autoimmune patients including systemic lupus erythematosus (SLE), rheumatoid arthritis (RA), scleroderma, autoimmune vasculitis, vitiligo, pemphigus and Wagner cases and 20 urine samples from healthy individuals were collected from Kerman Province in Southeastern Iran in 2010–2011. All urine samples were checked by KAtex after boiling for 5 min false positivity rate of the test was surveyed in different healthy and patients groups while boiling process was removed. Rheumatoid factor (RF) then was checked in sera of all cases to evaluate the relationship between RF and KAtex false positivity.

**Results:**

All samples represented negative results with KAtex when boiling was performed (100% specificity). Then, 20% positivity was evident in healthy cases. False-positive reactivity was more prominent observed in patient groups than healthy individuals, except in vitiligo. However, a significant difference was only observed in RA group (*P*<0.05). RF was related to KAtex false positivity.

**Conclusion:**

RA was described as the autoimmune disease in which KAtex false positivity was higher than normal population. RF or its metabolic products may have role in false positivity of KAtex but this finding needs to be confirmed by more reliable and improved experiments. Overall, immune system products should be considered in attempts for modification of KAtex for boiling process removal.

## Introduction

Visceral leishmaniasis (VL) is a notable public health concern in endemic regions of the world that usually is fatal if left untreated (1). VL has been reported from more than 65 countries, and the incidence of disease is estimated at 200000–400000 cases with 20000–40000 deaths annually (2). VL is also endemic in the Middle East countries including Iran. Important foci of VL and related hazard zones in Iran have been reported from the Northwest, Southwest and some Southern regions (3–8). *Leishmania infantum* is the main cause of VL in Iran although *L. tropica* as the causative agent of anthroponotic cutaneous leishmaniasis in urban regions including; Tehran, Shiraz, Mashhad, Kashan, Kerman and also some rural regions such as Bam and Birjand counties (9–13) was considered as the second causative agent of VL in Iran (5,14).

Primary diagnosis of VL is based on the history of travel to the endemic regions. The chief manifestations of the disease in these regions consist of irregular fever, weight loss, hepatosplenomegaly, lymphadenopathy, anemia and leucopenia (15). The gold standard test for diagnosis is microscopic detection of parasite in spleen or bone marrow macrophages. The risk of fatal hemorrhage and the need for expert personnel confined the spleen aspiration approach. The bone marrow aspiration is also a painful process with lower sensitivity rate (16, 17).

Hyperimmunoglobulinemia is a prominent immune response in VL in contrast to cutaneous leishmaniasis (CL) and mucocutaneous leishmaniasis (MCL) which induce weak humoral response and strong cellular immunity in the host (18). Therefore, different antibody detection tests have been evolved for diagnosis of VL which include indirect fluorescent antibody test (IFAT) as one of the most common method (19), ELISA as one of the most sensitive test and direct agglutination test (DAT) as a very highly specific, sensitive, also inexpensive and field-applicable diagnostic test (20,21). Antibody detection tests cannot generally discriminate between current and past infection. Cross-reaction response between different infective agents is also a limiting factor. KAtex is a newly designed latex agglutination test for detection of leishmanial antigens in urine of VL patients. Sensitivity and specificity of KAtex were reported as 65%–100% and 100%, respectively in the primary study (22). Study of KAtex by researchers in different endemic countries showed different sensitivities and specificities, respectively including 95.2% and 100% in Sudan, 73.9% and 82.4% in Ethiopia, 67% and 99% in India, 75% and 100% and 87% and 100% in two studies in Bangladesh, 57% and 90% and 47.7% and 98.7% in separate studies in Nepal, and 82.7% and 98.9% and 83.9% and 100% in two studies in Iran (23–30).

This test detects 5–20 kDa low molecular weight antigen in patient urine immediately after infection while the test result converts to negative following disease treatment (31). The main drawback of KAtex is false-positive reaction in some healthy or non-VL case samples. The false positivity can be removed by a 5-min boiling before testing. However, boiling is a bothersome step, which produces unpleasant odor in laboratory and confines the field applicability of the test due to needs for heater plate and related containers. Therefore, some efforts have been conducted to substitute boiling by other processes (32). In a study (unpublished) we have observed the high false positivity in urine of some autoimmune patients in comparison with healthy or non-auto immune cases.

This study was designed to survey false positive reaction of KAtex among autoimmune cases as compared with the healthy individuals.

## Materials and Methods

### Populations and Samples

The urine and sera samples were obtained from 20 healthy cases routinely referred to medical laboratories for check-up purposes and 92 autoimmune patients admitted in specialized hospitals in Kerman Province, Southeastern Iran in 2010–2011. Autoimmune cases consisted of systemic lupus erythematosus, rheumatoid arteritis, scleroderma, autoimmune vasculitis, vitiligo, pemphigus, and Wagner cases (Table 1). All the cases were collected from VL non-endemic regions. Their history was checked to reject cases with probable history of VL disease.

**Table 1:** The frequency of collected samples obtained from auto-immune and healthy individuals

**Status**	**NO.**	**Percent**
SLE	35	31.2
RA	20	17.9
Scleroderma	9	8
Auto-immune vasculitis	11	9.8
Vitiligo	6	5.4
Pemphigus	8	7.1
Wegner	3	2.7
Healthy	20	17.9
Total	112	100

### KAtex with Boiling

All urine samples have been checked by KAtex according to the manufacturers’ instruction (Kalon Biological/LTD, Guilford England) to exclude VL in healthy and autoimmune cases, reported100% and 98.9% specificity of the test in two studies (29, 30) in Iran.

### KAtex without Boiling

All samples were checked by KAtex while boiling process was ignored. Accordingly, the frozen patient urine samples were thawed at room temperature. Fifty-μl urine sample was added on the serological slide and positive and negative controls were used to evaluate the result of the test. Fifty μl of KAtex suspension was added to each sample and both controls for each run. Mixing was performed by gentle rotation on the rotator for 2 min. Finally, results were reported as negative, weakly positive (+−), positive (+), strongly positive (++), and very strong positive (+++ and ++++) based on the severity of agglutination.

### Rheumatoid Factor Test

The test was performed according to the manufacturer’s instruction (Human Tex RF).

### Ethics and Statistic Analysis

Written informed consents of the patients and healthy volunteers were obtained. The local Ethics Committee approved the study.

SPSS ver. 20 (Chicago, IL, USA) was used for data entry. Chi-square analysis was used for comparison of healthy and autoimmune groups. Significance level was at *P*<0.05. The relationship between RF and false positivity of KAtex was analyzed by McNemar test.

## Results

The proportion of females was higher for all the autoimmune diseases in comparison with the healthy group. Age distributions of autoimmune cases were classified into three levels: <20 yr old, 20–60 yr old, and >60 yr old, which were 10.9%, 70.6%, and 18.5%, respectively. SLE group showed the most cases with <20 yr old (17%) while pemphigus disease was revealed to have the most proportion of cases with higher 60-year-old ages (Table 2).

**Table 2:** Frequency of cases in different groups by sex and age

**Group**	**Sex**		**Age (yr)**		
	**Female**	**Male**	**< 20**	**20–60**	**> 60**
SLE	28	7	6	26	3
RA	15	5	1	13	6
Scleroderma	6	3	1	7	1
Auto-immune vasculitis	7	4	1	8	2
Vitiligo	4	2	1	4	1
Pemphigus	5	3	0	4	4
Wagner	2	1	0	3	0
Healthy	55	45	5	11	4

All healthy and autoimmune cases were shown to be negative for KAtex when urine samples were boiled. The result of KAtex without boiling process was different for various groups of patients in comparison with healthy individuals. Four out of 20 cases in healthy group showed false positive KAtex result. Twenty out of 35 SLE cases also displayed a false positive KAtex result. There was no significant difference between SLE and healthy groups in this regard. Out of 20 RA cases, only nine patients showed negative KAtex result. The comparison between RA cases and the healthy group demonstrated a significant difference (*P*<0.05).

Four out of 11 autoimmune vasculitis cases had false positive KAtex result, although no significant difference was indicated in comparison with the healthy group. Pemphigus and vitiligo groups also showed no significant results compared with the healthy control group. Wenger disease cases were not analyzed due to low numbers. Severity of agglutination reaction was different for each group. RA showed the most one-plus false positive results comparing with other autoimmune disease groups. The only two-plus agglutination reaction was shown for an autoimmune vasculitis case. Table 3 shows chi-square test results for autoimmune groups compared with control group and the severity of agglutination for different groups.

**Table 3:** Chi-square test results for autoimmune groups compared with control group and the severity of agglutination for different groups

**Status**	**KAtex positive**	**Percent**	***P* value**	**+−**	**+**	**++**	**Total**
SLE	12	34.3%	0.359	7	5	0	35
RA	11	55%	0.022	5	6	0	20
Scleroderma	2	22.2%	1.000	1	1	0	9
Auto-immune vasculitis	4	36.4%	0.405	1	2	1	11
Vitiligo	1	16.7%	1.000	1	0	0	6
Pemphigus	2	25%	1.000	1	1	0	8
Wegner	1	33.3%	-	0	1	0	3
Healthy	4	20%		2	2	0	20

RF was checked for all healthy and autoimmune cases. Nineteen out of 20 RA cases, 4 out of 35 SLE cases, one out of six pemphigus and one out of 11 autoimmune vasculitis cases showed positive results. All other samples were negative for RF test. KAtex result was (false) positive for two out of four RF positive SLE cases, but both autoimmune vasculitis and pemphigus RF positive cases had negative KAtex response. The only RF-negative RA case had negative KAtex result.

Overall, 13 cases of 37 KAtex positive cases, revealed RF positive test (35.1%) while only 12 out of 75 KAtex negative cases (16%) demonstrated RF in their sera. The relationship between RF and false positivity of KAtex was shown in which no difference was observed between RF test and KAtex (without boiling) results by McNemar test (*P*=0.065).

## Discussion

Latex agglutination test for kala-azar was presented in Liverpool Tropical Medicine Faculty in England in 2001 (22). This test has been considered by researchers due to some profits including easy procedure, inexpensiveness, field applicability of the test, also very high specificity, and acceptable sensitivity. These properties made the test as a suitable VL diagnostic tool especially in less developed countries and in the regions far from referral centers (31). KAtex is also the only available antigen detection testing kit can be used for following the treatment of kala-azar (33). The main drawback of KAtex is false positivity of the test without boiling process and the cause of which remained unknown.

Regarding our result, false positivity of KAtex without boiling was higher than control (healthy) group for all autoimmune groups except for vitiligo, although only RA group showed significant difference. RA is an autoimmune disease, diagnosed by para-clinically positivity of rheumatoid factor in patient’s sera. Rate of positivity of RF was higher among KAtex (without boiling) positive cases rather than other cases and relationship was shown between RF and KAtex false positivity. This finding showed probable role of RF in false positivity of KAtex. RF is an antibody produced against fragment crystallizable (Fc) fraction of IgG in RA and some other autoimmune cases sera. Furthermore, RF is produced in some other diseases such as leukemia. Totally, 1%–5% of healthy peoples have also revealed positive RF test (34). The rate of RF positivity rises with increase of age. Positive RF test has also been shown in some bacterial, viral, and parasitological diseases. Some parasitological diseases with RF positive results are malaria, chagas, trypanosomiasis, and schistosomiasis.

Recently, various studies confirmed the production of RF in kala-azar patients. RF has been supposed to play a role in protection of host against these infections (35–39). RF could result in the false positivity of rapid test for malaria (RTD) (40, 41). RF or other autoimmune antibodies were also offered as the probable cause of false positivity of toxoplasmosis IgM testing (42). Different factors and antibodies are produced by immune system regarding inflammatory basis of autoimmune or infectious disease. Secretion of these compounds such RF or their bi-products from kidneys with normal or impaired function may result in cross-reaction in diagnostic test on urine samples. Therefore, either rheumatoid factor and its metabolic products or other unknown factors that produced and secreted in urine of RA cases may play a role in false positive reactivity of KAtex.

A previous study in Iran (30) on the confirmed VL cases showed strongly (++) and very strongly positive (+++ and ++++) agglutination for more than 41% of urine samples while only near to 23% showed weakly positive result (unpublished data). However, in the current study, more than 48% (18 out of 37) of all positive autoimmune cases and also 45.5% of positive RA cases showed weakly positive agglutination while strong positivity was presented in only one sample and very strong agglutination was not observed for any autoimmune cases when boiling process omitted. Therefore, although, higher positive rate has been shown for autoimmune disease rather than healthy control but in comparison with VL cases severity of agglutination was lower. All over, regarding semi-quantitative nature and high subjectivity of KAtex (43), severity of agglutination should be considered in every attempt for improvement and modification of the test. In addition, strongly positive KAtex results for VL cases when boiling facilities are not available may partly be reliable. On the other hand, difference in sensitivity of the test in different studies and in different endemic regions in the world may be reflected in the role of individual assessment of weakly positive results by laboratory staffs. Demographic data obtained from our study showed the higher number of female autoimmune patients. This data reconfirmed the higher rate of autoimmune disease in females compared to males.

## Conclusion

RA was described as the autoimmune disease in which KAtex false positivity was higher than normal population. RF factor was related to false positivity of KAtex and may have a role in false positivity of the test but this finding needs to be confirmed by more reliable and improved experiments. Overall, immune system products should be considered in attempts for modification of KAtex in boiling process removal.

## Ethical considerations

Ethical issues (Including plagiarism, informed consent, misconduct, data fabrication and/or falsification, double publication and/or submission, redundancy, etc.) have been completely observed by the authors.
